# Women Leaders and Pandemic Performance: A Spurious Correlation

**DOI:** 10.1017/S1743923X20000525

**Published:** 2020-07-30

**Authors:** Jennifer M. Piscopo

**Affiliations:** Occidental College

**Keywords:** Women, chief executives, coronavirus, pandemic, state capacity

## Abstract

The connection between women leaders and superior pandemic performance is likely spurious. This narrative overlooks that women currently govern precisely the kinds of countries that should mount effective pandemic responses: wealthy democracies with high state capacity. This article maps where women currently serve as presidents and prime ministers. The article then uses data from the Varieties of Democracy Project and the Organisation for Economic Co-operation and Development to show that many women-led countries score high on state capacity and that high-capacity states have low coronavirus mortality regardless of whether they are led by women or by men. Arguments emphasizing women chief executives’ superior pandemic performance, while offered in good faith, are misleading.

By many metrics, Presidents Donald Trump in the United States and Jair Bolsonaro in Brazil bungled their governments’ coronavirus responses. Trump and Bolsonaro have manipulated information about case numbers, minimized the public health threat, and promoted dangerous drug therapies (Osborn 2020; Paz 2020). By contrast, German chancellor Angela Merkel and New Zealand prime minister Jacinda Ardern closed borders and initiated widespread testing, lowering cases and mortality. As of June 1, 2020, Germany had 10 deaths per 100,000 people and New Zealand had reported 22 deaths *overall*.^1^ Such contrasts led pundits to champion a gender effect, with articles praising women leaders’ superior pandemic performance appearing in CNN, *Forbes*, the *Guardian*, the *Harvard Business Review*, the *New York Times*, Politico, and the *Washington Post*, among other outlets.

Yet this argument overlooks the kinds of countries that women presidents and prime ministers currently lead: primarily global North countries, meaning wealthy, liberal democracies with high state capacity and therefore good governance. These factors theoretically also facilitate countries’ ability to mount effective pandemic responses. The concentration of women chief executives in the global North means that a correlation between women leaders and superior pandemic performance is therefore likely spurious. Rather than leader gender driving pandemic performance, a third variable—high state capacity—corresponds with both leader gender and containing the pandemic.

In this article, I use the Varieties of Democracy (V-Dem) data (Coppedge et al. 2020) and the Organisation for Economic Co-operation and Development's social cohesion indicators (OECD 2019) to establish two points. First, the European and Scandinavian countries that today elect women also rank high in measures of state capacity: they have transparent laws, impartial public administrations, high trust in government, low perceived corruption, high social spending, and high life satisfaction. Second, among high-capacity countries, those led by women and those led by men have comparable coronavirus outcomes. These patterns complicate straightforward connections between leader gender and pandemic containment.

## WOMEN CHIEF EXECUTIVES IN 2020

Currently, 12 women serve as chief executives, either alone or as leaders of government in a dual executive system (Catalyst 2020): nine women govern in the global North compared with three in the global South (Table 1). Not counted are women serving interim terms, since neither they nor their party were chosen by popular election.^2^ For example, Bolivia's November 2019 political crisis removed the male president and vice president and left Senator Jeanine Áñez leading a caretaker government. Also not counted are the 10 women holding the less prestigious position in a dual executive, in countries as diverse as Nepal, Singapore, and Georgia. Finally, Simonetta Sommaruga presides over the Swiss Federal Council, a first among equals position that conveys no additional policy-making powers. Though women serving in these lesser chief executive roles are often well-known career politicians, their formal powers are relatively few (Jalalzai 2008).

**Table 1.** Women heads of government emerging from popular elections, by global North and South

Name	Country

Jacinda Ardern	New Zealand
Ana Brnabić	Serbia
Mette Frederiksen	Denmark
Tsai Ing-wen	Taiwan
Katrin Jakobsdóttir	Iceland
Sanna Marin	Finland
Angela Merkel	Germany
Erna Solberg	Norway
Sophie Wilmès	Belgium
Sheikh Hasina	Bangladesh
Silveria Jacobs	Sint Maarten
Mia Mottley	Barbados

*Source: Catalyst (2020)*, updated by author.

The global North/global South division reflects countries’ relative power and privilege within the international system. Global North countries are developed, wealthy, and usually politically and economically liberal (Müller 2020). Of the nine global North places with women leaders, all except Serbia score at or above .7 (where 1 is most democratic) on V-Dem's polyarchy and liberal democracy measures.^3^ Even with debate over certain countries’ categorization as “North” or “South” (Müller 2020), the world has fewer global North than global South countries, making the current concentration of women leaders in the North especially noteworthy.

This pattern contrasts with one or two decades ago, when women presidents and prime ministers predominated in the global South, especially South Asia, Southeast Asia, and Latin America (Jalalzai 2008). Earlier research observed that women attained chief executive posts more frequently in countries with high instability and low political institutionalization (Jalalzai 2008), that were less wealthy (Thames and Williams 2013), and that had highly gender-segregated societies (O'Brien and Reyes-Housholder 2020). Yet Europe and Scandinavia do not fit this profile. Today's women leaders largely do not govern the poor or weakly institutionalized countries of the global South but the high-capacity states of the global North.

## A SPURIOUS CORRELATION

My argument has two points: (1) today's women leaders are concentrated in “high-capacity countries,” and (2) high-capacity countries led by women and high-capacity countries led by men both have contained the pandemic relatively well. I draw my sample from the OECD's Social Cohesion Report (OECD 2019).^4^ The report includes seven women-led countries from the global North: Belgium, Denmark, Iceland, Finland, Germany, New Zealand, and Norway. With seven women executives and 38 countries total, the sample size is too small for full regression analysis, but it can indicate patterns.

Scholars tie state capacity to good governance, with both concepts tapping into countries’ ability to perform tasks and exercise power effectively, efficiently, and fairly (Charron, Lapuente, and Rothstein 2013; Joshi 2011). How to conceptualize and measure state capacity remains debated (Charron, Lapuente, and Rothstein 2013). I cannot resolve that debate here, but I select six indicators based on face validity and data availability: transparent laws with predictable enforcement, impartial public administrations, trust in government, perceived corruption, social spending, and overall life satisfaction. Transparent and impartial governments should issue reliable scientific advice. When citizens follow that advice—such as staying home and wearing masks—mortality should decline. Yet following governments’ advice requires that citizens view government as acting in their best interests, directly captured by trust, perceived corruption, and social spending, and indirectly captured by life satisfaction.

Transparent laws and impartial administrations are 2019 V-Dem indicators (Coppedge et al. 2020), measured via expert ratings on a 4-point scale where 4 is the most transparent or most impartial. The remaining OECD (2019) indicators are measured as follows: the proportion of survey respondents reporting trust in government and perceived government corruption in 2017; social spending (including pensions and health) as a percentage of gross domestic product in 2018; and survey respondents’ life satisfaction on a 10-point scale, where 10 is the most satisfied, in 2017. To measure pandemic performance, I use coronavirus deaths per 100,000 people as of June 1, 2020.

Table 2 reports how the women-led OECD countries, men-led OECD countries, and all OECD countries compare on the state capacity measures and on COVID-19 mortality rates. (For perceived corruption, lower numbers indicate better ratings.) The results underscore the concentration of women leaders in high-capacity countries, as OECD countries with women leaders have higher capacity scores compared with the OECD average and with OECD countries with men leaders. Women chief executives are found in countries that have more transparent laws, more impartial public administrations, higher trust in government, lower perceptions of corruption, higher social spending, and greater life satisfaction.

**Table 2.** Comparing state capacity for women- and men-led countries in the OECD

Indicator	OECD Average	Women-Led OECD Average	Men-Led OECD Average	Women-Led vs. Men-Led, One-Tailed	Women-Led vs. Men-Led, Two-Tailed

Transparent laws	3.21	3.86	3.06	*p* = .006	*p* = .013
Impartial administrations	3.34	4	3.19	*p* = .013	*p* = .026
Trust	43.3	53.3	40.9	*p* = .028	*p* = .056
Perceived corruption	55.6	34.4	60.7	*p* = .003	*p* = .006
Social spending	20.0	24.4	19.0	*p* = .01	*p* = .02
Life satisfaction	6.62	7.37	6.46	*p* = .0006	*p* = .001
COVID-19 mortality	16.6	15.9	16.5	*p* = .53	*p* = .94

Moreover, these differences are statistically significant, though the small sample size urges caution. Table 2 reports difference of means tests between women-led countries and men-led countries, including a one-tailed test for women-led countries’ higher scores and a two-tailed test for the absolute values.^5^ For both one-tailed and two-tailed tests, the differences are statistically significant at the 1 or 5 percent level (except the two-tailed test for trust, which is statistically significant at the 10 percent level). These results provide initial, suggestive support for the claim that high-capacity countries also happen to elect women leaders.

Turning to coronavirus mortality, Table 2 also shows that women-led countries have, on average, fewer deaths than men-led countries: 15.9 deaths per 100,000 people, compared with 16.5 deaths per 100,000 people. This result could confirm pundits’ suspicions, but the difference of means tests are *not* statistically significant.

Further, mortality rates are comparable across high-capacity countries, no matter leaders’ gender. I examine all OECD countries that perform above the OECD average on five or all six capacity measures. This approach yields 12 high-capacity countries: Switzerland, with its collegial federal council; six countries led by women (Iceland drops out, given its relatively poor scores on trust and perceived corruption), and five countries led by men (Australia, Ireland, Luxembourg, the Netherlands, and Sweden). Per 100,000 people, the six women-led high-capacity countries have an average mortality rate of 18.6 deaths with a standard deviation of 12.6 deaths, and the five men-led high-capacity countries have an average mortality rate of 27.9 deaths with a standard deviation of 7.4 deaths. Men-led countries average about nine more deaths, but women-led countries have more variable death rates. The difference of means are not statistically significant (*p* = .72 for women > men, *p* = .56 for absolute values; *p* = .28 for men > women), though the very small sample size again urges caution. Additionally, both 18.6 and 27.9 deaths per 100,000 people are relatively low mortality rates. As a comparison, Spain and the United Kingdom—two OECD countries widely viewed as mismanaging their pandemic responses (Henley 2020; Tremlett 2020)—each had about 57 deaths per 100,000 people.

These patterns cohere with other analyses. Bosancianu et al. (2020) find that institutional trust and bureaucratic capacity lower death rates throughout the pandemic, while corruption raises death rates as the pandemic lengthens. They find no relationship between women leaders and deaths. Similarly, Shay (2020) finds no gender effect for when U.S. governors issued shelter-in-place orders. Women *and* men Democratic governors acted early, and governor party corresponds with similar institutional factors, such as trusting experts and institutions (Piscopo 2020).

## WOMEN LEADERS IN DIFFICULT TIMES

Commentators championing women chief executives’ pandemic performance largely overlook a common factor that corresponds with both electing women and managing pandemics well: having good governance. I offer suggestive evidence to support this claim, but definitive econometric accounts linking state capacity to women leaders’ selection and coronavirus mortality are beyond this short article's scope. This initial analysis highlights women chief executives’ current concentration in the global North, the exact kinds of countries theoretically well-positioned to mount effective pandemic responses.

A spurious correlation does not mean leaders’ gender bears no consequence for pandemic performance. Indeed, women leaders’ greater social concern may lead them to *increase* state capacity, perhaps by expanding pandemic-related social spending (Funk 2020). More broadly, the connection between leader gender and pandemic performance upends the traditional association between chief executive office, masculinity, and effectiveness (O'Brien and Reyes-Housholder 2020). Ardern and Merkel confound gendered expectations about women's fragility, acting with level-headedness while Bolsonaro and Trump bluster futilely. Moreover, women leaders combine this unflappability with stereotypically feminine traits such as empathy, perhaps transforming how the public evaluates women chief executives (Johnson and Williams 2020).

Yet this narrative—offered in good faith to garner support for women's leadership—may backfire. The pandemic has raised women leaders’ profiles, but gendered double standards remain. Women governing during crisis face shorter tenures, harsher exits, and disproportionate blame compared with similarly situated men (O'Neill, Pruysers, and Stewart 2019; Reyes-Housholder 2019; Thomas 2018). Making women leaders into icons of coronavirus containment could heighten voters’ dissatisfaction with their performance as the pandemic lingers and even worsens.

## Footnotes

1. Mortality rates are calculated using the University of Washington's death counts (https://covid19.healthdata.org/) and United Nations World Population Prospects population data in millions (https://population.un.org/wpp/).

2. Thames and Williams (2013) also separate non-interim leaders from interim leaders.

3. Taiwan's polyarchy score is .697.

4. The OECD data exclude member Colombia but include nonmembers Russia and Brazil. The complete member list can be found at https://www.oecd.org/about/members-and-partners/.

5. Since lower values mean less perceived corruption, the one-tailed test is women < men (for others, it is women > men).
